# Functional Detection of TNF Receptor Family Members by Affinity-Labeled Ligands

**DOI:** 10.1038/s41598-017-06343-4

**Published:** 2017-07-31

**Authors:** Yang Xu, Lingmo Chang, Anliang Huang, Xiaojun Liu, Xinyu Liu, Hong Zhou, Joshua G. Liang, Peng Liang

**Affiliations:** 1 Department of Biochemistry & Molecular Biology, College of Life Sciences, Sichuan University, Chengdu, 610064 China; 2 Laboratory for Gene and Cell Therapy, Sichuan University, Chengdu, 610064 China; 3 Department of Biochemistry and Molecular Biology, Southwest Medical University, Luzhou, 646000 China; 4 Clover Biopharmaceuticals, Chengdu, 610041 China

## Abstract

Aberrant expression of TNF family of cytokines has been linked to human diseases, and biologics targeting their signaling have become the best selling drugs globally. However, functional detection with labeled ligands for accurate detection of TNFR family of receptor-expressing target tissues or cell types remains to be developed. Here we show that TNF receptor family members are heat-stable and can be recognized both *in vitro* and *in vivo* by their ligands labeled with alkaline phosphatase. Such an approach may be used in lieu of antibodies for the identification of the cell types involved in receptor signaling during disease onset and progression.

## Introduction

The tumor necrosis factor (TNF) superfamily (TNFSF) of cytokines and TNF receptor superfamily (TNFRSF) display diverse physiological functions and play critical roles in the development and homeostasis of the immune-, nervous- and musculoskeletal-systems in mammals^1^. TNFSF consists of 19 known ligands that contain the extracellular TNF homology domain (THD) and are all initially expressed as type II transmembrane proteins, although most can exist also in soluble form after extracellular domain cleavage by proteolysis^1,2^. These ligands signal through 29 structurally related type I transmembrane receptor proteins of TNFRSF containing the extracellular cysteine-rich domain (CRD)^1,3^.

Abnormal expression of TNF family cytokines or their receptors has been linked to a host of major human diseases including arthritis, psoriasis, osteoporosis and cancer. Elevated localized expression of TNFα has been shown to be one of the underlying causes for various autoimmune and inflammatory disorders such as psoriasis and arthritis^1,4^. While biologic therapies blocking TNFα currently represent the largest-selling class of blockbuster drugs globally^5^, the underlying causes of TNFα signaling in disease onset and progression as well as resistance to anti-TNFα therapy in some patients remain obscure^6,7^. Moreover, TNF-related apoptosis-inducing ligand (TRAIL) has been shown to potently induce apoptosis in a tumor-specific fashion against multiple human cancer cell lines from various tissue origins both *in vitro* and *in vivo*, and once hailed as a promising magic bullet against cancer^8,9^. Despite of very encouraging initial data coming of Phase I clinical trials, recombinant TRAIL (Apo2L) as well as its receptor agonist monoclonal antibodies failed to hold up the expectation in later trials^10^. The failure could be due to the lack of consideration of the expression of death receptors (DR4 and DR5) on tumor cells while selecting disease indications and patients^11^. Thus a more accurate identification of the tissues and cell types expressing receptors of TNFRSF both *in vitro* and *in vivo*, particularly during disease pathogenesis and progression can be enlightening to understanding molecular mechanisms underlying cytokine networks involved in inflammation and cell programmed death. Such knowledge may lead to better diagnosis and more targeted treatment of the diseases involved.

However, current methodologies for detection of TNFRSF are largely limited to antibody-based approaches that without proper controls could be error-prone due to potential non-specificities of primary / secondary antibodies and labels used^12^.

Alkaline phosphatase (AP)-tagged ligands are a useful tool for receptor detection and have been used to discover a number of important cell surface receptors and ligands, including receptors for leptin^13^ and IL-24^14^ as well as ligands for Kit, Mek4 and Sek receptor tyrosine kinases^15,16^. Although the C-terminal AP-tagged full-length TNFα had been used to track the shedding of soluble TNFα from the type II membrane-bound form on the cell membrane^17,18^, N-terminal labeling of soluble TNF family of cytokines with AP-Tag for receptor detection has not been reported.

In this brief communication, we demonstrate that N-terminal AP-tagged soluble TNFSF cytokines – AP-TNFα, AP-TRAIL and AP-RANKL – can be readily produced as secreted proteins from Chinese hamster ovary (CHO) cells, and used as probes based on the AP activity to detect their respective target receptors both *in* *vitro* and *in* *vivo*. In particular, we show that TNF receptor family members are extremely heat stable, which makes it possible for their accurate detection under both native and denaturing conditions.

## Results

**Figure 1:**
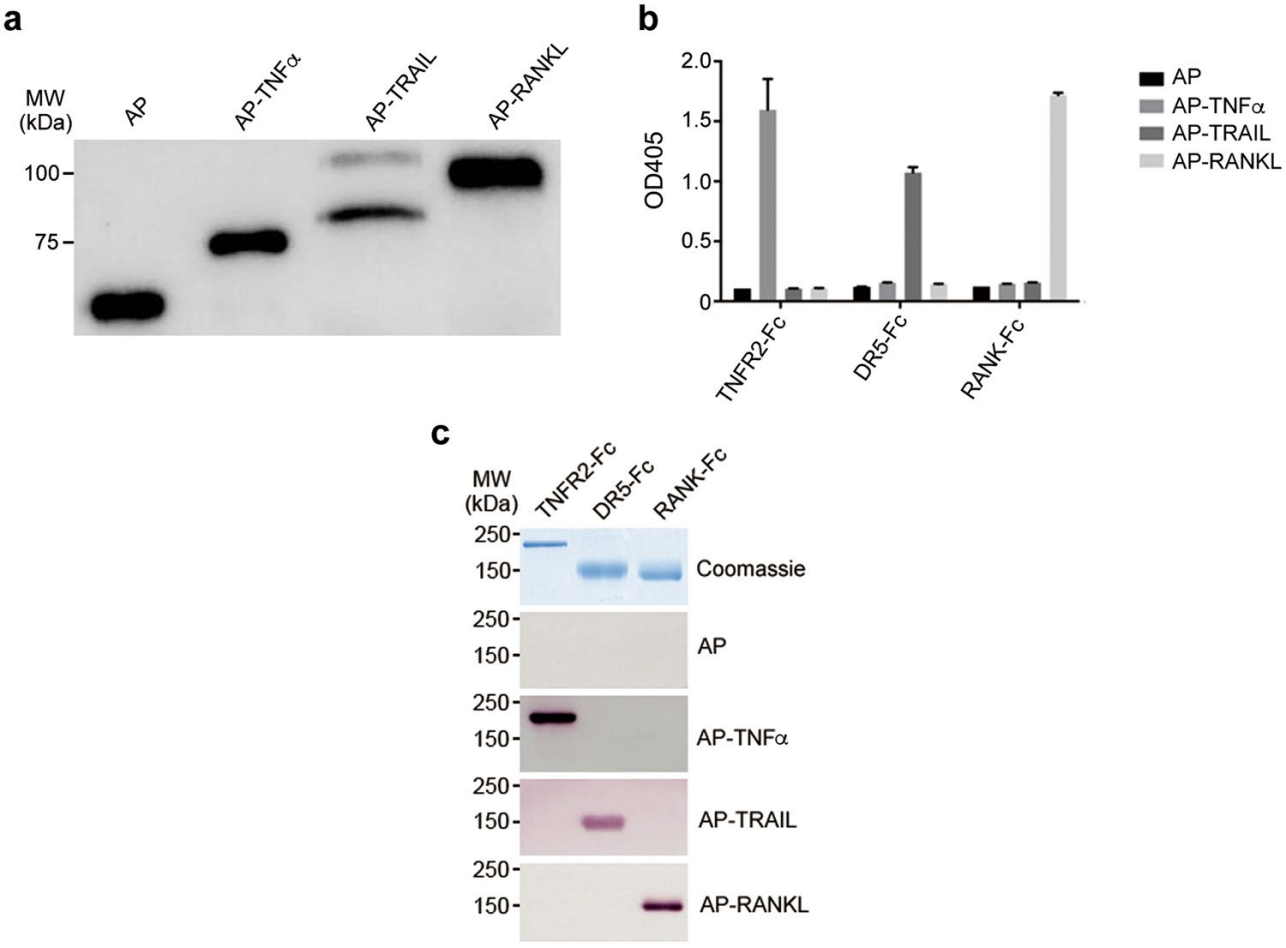
Alkaline phosphatase (AP)-tagged TNFSF ligands each bind specifically to their respective receptors *in vitro*. (**a**) Conditioned media from CHO cells expressing AP, AP-TNFα, AP-TRAIL and AP-RANKL were analyzed by Western blot using polyclonal antibody against AP to confirm the size and integrity of the fusion proteins. (**b**) ELISA assays for binding of AP-tagged TNFSF ligands to recombinant TNFR2-Fc, DR5-Fc and RANK-Fc fusion proteins. Equal concentration AP activity (1 U/mL) of the above mentioned AP-tagged ligands were used as probes, with AP alone as a negative control. (**c**) Affinity blotting analysis of TNFR2-Fc, DR5-Fc and RANK-Fc receptor fusion proteins with AP-tagged TNFSF ligands. The purified TNFR2-Fc, DR5-Fc and RANK-Fc receptor fusion proteins were separated on a denaturing SDS-PAGE under non-reducing condition, transferred to a PVDF membrane and probed with the AP-tagged ligands as indicated, with AP alone as a negative control and Coomassie Blue staining for loading control.

Since TNF family of cytokines are initially made as type II membrane proteins with their C-termini protruding on the cell surface, we decided to tag the N-termini of the soluble forms of these cytokines with human placental alkaline phosphatase (AP) using AP-Tag technology. Guided by the signal peptide sequence from the AP, we showed that three members of AP-tagged TNF family of cytokines, AP-TNFα, AP-TRAIL and AP-RANKL could be made at high level as secreted proteins from Chinese hamster ovary (CHO) cells, with AP activity ranging from 15–60 U/mL and readily detectable by Western blot analysis (Fig. 1a). These AP-tagged ligands not only manifested excellent receptor binding specificity on an ELISA plate coated with their respective target soluble receptor-Fc fusion proteins (Fig. 1b), but also could be used much as antibodies for the affinity detection of their corresponding soluble receptor-Fc fusion proteins on Western blots under non-reducing condition (Fig. 1c**)**. The fact that these receptor fusion proteins had been heat denatured and separated on an SDS-PAGE and transferred to a PVDF membrane could be still be recognized by their respective AP-tagged ligands suggested that members from TNFRSF are heat-stable with likely linear epitope for ligand recognition.

**Figure 2:**
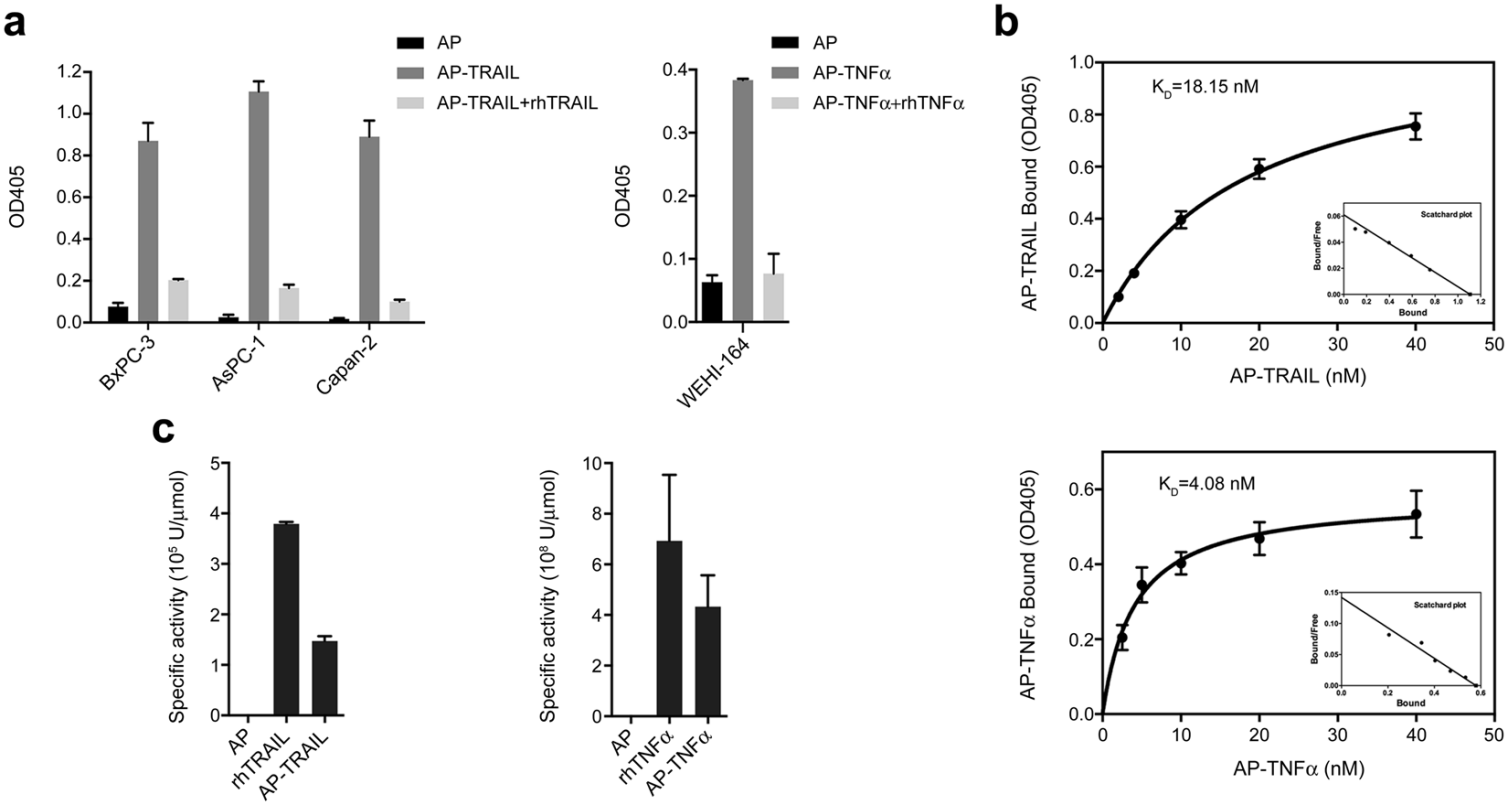
Cell surface receptor binding of alkaline phosphatase (AP)-tagged TNFSF ligands. (**a**) Detection of cell surface receptor(s) from either cultured human pancreatic cancer cell lines (BxPC-3, AsPC-1, and Capan-2) with AP-TRAIL (left) or WEHI- 164 cells with AP-TNFα (right). AP alone served as a negative control, while 100-fold excess of unlabeled rhTRAIL or rhTNFα served as controls for receptor binding specificity. (**b**) Saturation binding kinetics of AP-TRAIL to BxPC-3 cells (top) and AP-TNFα to WEHI-164 cells (bottom) were determined with increasing concentration of the AP-tagged ligands. The data presented as Scatchard plots were shown as insets in the bottom right of saturation binding curves. (**c**) Analysis of the biological activities AP-tagged TRAIL and TNFα in comparison to untagged ligands by bioassays using TRAIL-sensitive BxPC-3 and TNFα-sensitive WEHI-164 cells, respectively as described above.

Next, we examined the ability of AP-tagged ligands to detect their corresponding cell surface receptors from cultured cell lines. Due to the observation that some human cancer cell lines, including AsPC-1 (human pancreas adenocarcinoma ascites metastases), DLD-1 (human colorectal adenocarcinoma) and Capan-2 (human pancreatic ductal adenocarcinoma), exhibit high level of endogenous AP activity which cannot be inactivated by at 65 °C usually used for removing background AP activity from host cells (data not shown), we examined the binding of AP-TRAIL to cultured human pancreas adenocarcinoma cell lines after heat treatment of the cells at 100 °C for 10 min. We found that the endogenous AP activity was essentially eliminated, while binding of AP-TRAIL to the cell surface death receptors (DRs) on cancer cells was preserved (Fig. 2a, left). Similarly, we found that WEHI- 164 cells – a well established cell line used for bioassays of TNFα^19^ – were able to bind to AP-TNFα with minimal background AP activity after heat inactivation of endogenous AP in boiling water (Fig. 2a, right). The receptor binding specificities were confirmed by the significant reduction in signals from AP-tagged ligands with excessive unlabeled rhTRAIL and rhTNFα, respectively (Fig. 2a). These findings are consistent with results from receptor binding *in vitro* (Fig. 1c), indicating that TNFRSF of proteins are heat stable. It has been previously shown that some membrane proteins are thermostable^20–22^; however, this characteristic which appears to be shared across TNFRSF members has not been previously investigated. Missense mutations have been identified in thermostable mutants of the diacylglycerol kinase and soluble enzyme *p-nitrobenzyl* esterase^20,21^, and given our observation that TNFR2-Fc fusion protein under reducing conditions cannot be recognized by its ligand (data not shown), suggesting that primary and secondary protein structures may play a critical role in ligand recognition.

A typical ligand-receptor binding is expected to be saturable with increasing ligand concentration. This was indeed the case for both AP-TRAIL and AP-TNFα, both of which showed saturation receptor binding kinetics to BxPC-3 and WEHI-164 cells with Kd being 18.15 nM and 4.08 nM, respectively (Fig. 2b). In addition, as expected, the AP-tagged TRAIL and TNFα fusion proteins retained significant level of biological activities as determined by their ability to induce apoptosis for BxPC-3 and WEHI-164 cells, respectively, while AP alone could not (Fig. 2c).

**Figure 3:**
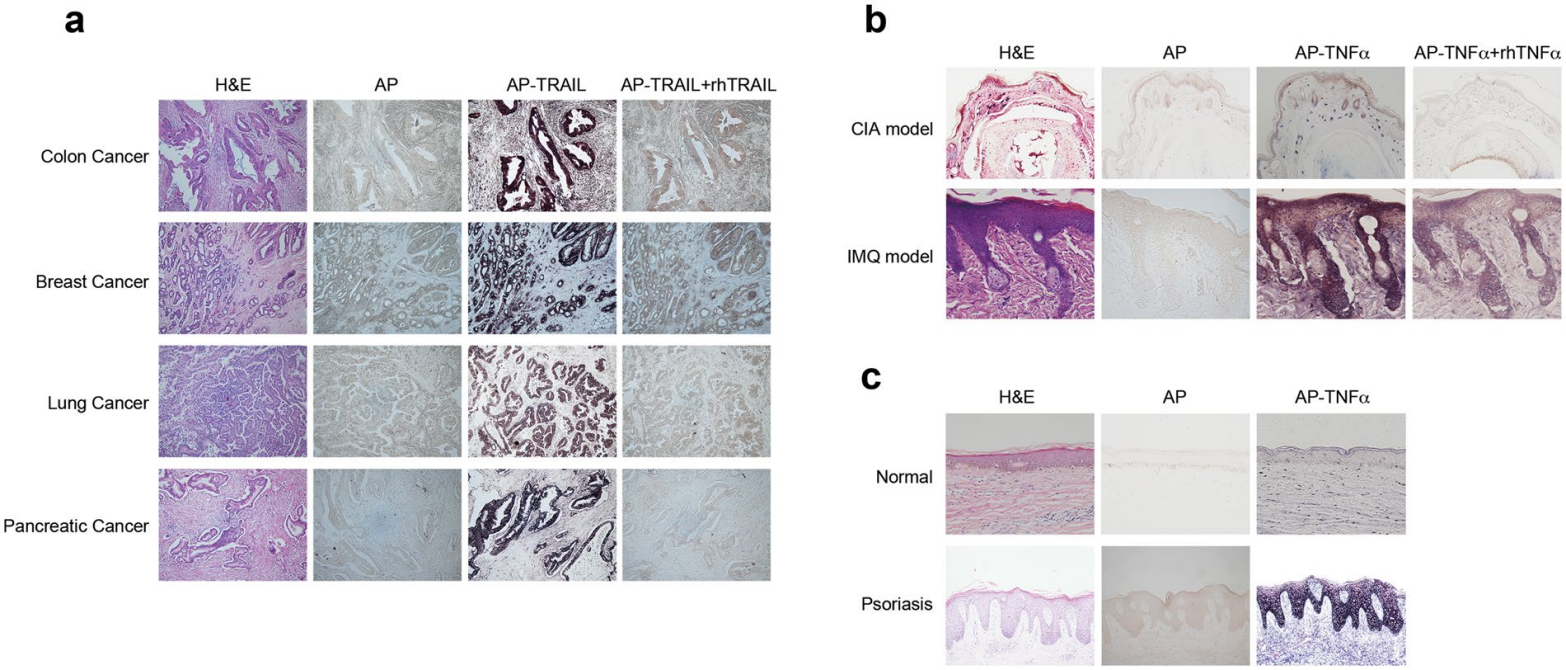
Detection of receptors for TRAIL and TNFα in paraffin-embedded tissue sections. (**a**) Detection of death receptor(s) or decoy receptors for TRAIL in paraffin-embedded human cancer specimens using AP-TRAIL. Η&Ε and AP alone staining served as controls, AP-TRAIL staining with 10-fold excess of unlabeled rhTRAIL served as a control for receptor binding specificity. (**b**) Detection of abnormal expression of TNFR in paraffin-embedded specimens from either mouse CIA (hind paws) or IMQ model (skins) with AP-TNFα. Η&Ε and AP alone staining served as controls. AP-TNFα staining with 10-fold excess of rhTNFα served as a control for receptor binding specificity. (**c**) Detection of abnormal expression of TNFR in paraffin-embedded specimens of human psoriasis in comparison with normal human skin tissue with AP-TNFα. Η&Ε and AP alone staining served as controls.

To further explore the utility of AP-tagged ligands from TNFSF in functional detection of their corresponding receptor expression *in vivo*, in particular in paraffin-embedded tissue sections, we then examined DRs expression in a panel of human cancer tissues. Paraffin-embedded specimens from human colon, breast, lung, and pancreatic cancers were de-waxed, sequentially rehydrated with ethanol and then probed with AP-TRAIL. The results observed were striking, showing high level of cancer epithelial cell-specific DRs expression in certain population of all four cancer types analyzed (Fig. 3a). AP alone served as a negative control for non-specific receptor binding, indicating that AP-TRAIL binding was specific and that endogenous AP activity did not interfere with the tissue staining. Furthermore, in all four human cancer types analyzed, signals from AP-TRAIL binding could be essentially completely competed away with 10-fold excess of untagged rhTRAIL suggesting that the DR signal detected by AP-TRAIL is indeed ligand specific. It should be noted that among the cancer patient specimens that we analyzed so far, high-level DRs expression shown as in Fig. 2c were among the minority, accounting for fewer than 15% of all samples analyzed. Given that TRAIL signals through DRs via extrinsic cell death pathway, past failure in clinical trials of using Apo2L or DRs agonist mAbs in the treatment of human cancer could be due to the lack knowledge of DRs overexpression in cancer population selected. Thus, the use of DRs expression level as a marker using AP-TRAIL may be tested in future trials, which may increase the chance of drug response for TRAIL (Apo2L) or the DRs agonist mAbs.

Finally, we also explored AP-TNFα as a probe in tissue staining of paraffin-embedded sections from both collagen induced arthritis (CIA) and imiquimod (IMQ) –induced psoriasis disease models in mice (Fig. 3b). As expected, we observed sensitive and ligand-specific functional detection of TNFR in tissues from both disease models where TNFα have been implicated in the pathogenesis. AP-TNFα binding to tissue in the CIA model was localized to what appeared to be immune infiltrate cells, which we later confirmed and pinpointed as macrophages^23^. On the other hand, AP-TNFα binding to tissue sections in the IMQ-induced psoriasis model indicates that its receptors are quite ubiquitously and highly expressed throughout the disease-affected epidermis. AP alone and competition with unlabeled TNFα served as controls for receptor binding specificity. With this method, we further analyzed human biopsy skin sections from both psoriasis patients and normal subjects. The result revealed that certain population of psoriasis patients exhibited striking over-expression of TNFR in keratinocytes, in comparison with the epidermis from normal skins (Fig. 3c). This result is consistent with previous finding in a systematic analysis of TNFR expression from human psoriasis using TNFRI and TNFRII specific antibodies^24^. From these *in situ* analysis of TNFR expression, it is interesting to note that while TNF antagonists such as soluble TNFRII-Fc fusion protein (Enbrel) and anti-TNFα mAbs have become main stakes in the treatment of autoimmune diseases, the involvement of TNFR expressing cell types seemed to be strikingly different, with immune infiltrates in RA and keratinocytes in psoriasis being the major source of cell types over-expressing TNF receptors.

## Discussion

In this study we demonstrated that AP-tagged TNFSF cytokines can be used as probes for accurate functional detection of TNFRSF expression *in situ* both *in vitro* and *in vivo* from disease tissues of animal models and human patients. The simplicity, specificity and functional-binding of AP-tagged TNFSF ligands to TNFRSF may make it a preferable approach compared to standard immunohistochemistry and FACS analysis utilizing antibodies. Such antibody-based approaches without proper controls can be error-prone due to issues of specificities of primary / secondary antibodies and labels used^12^, whereas the use of AP-tagged ligands as probes preserves natural ligand-receptor recognition, requires fewer intermediary steps for receptor detection. Another advantage is that a single AP-tagged TNFSF ligand can detect the presence of all its corresponding receptors; for example, AP-TNFα can bind to both TNFRI and TNFRII, whereas an antibody approach would require two separate primary antibodies recognizing each receptor type. Conceivably, AP-tagged TNFSF ligands could also lead to the discovery of novel receptors or previously unknown cell-types expressing TNFRSF proteins, if used in tandem with standard immunohistochemistry- based approaches.

Importantly, the fundamental involvement of TNF ligand-receptor pathways and their precise coordination with other signaling pathways in human diseases remains to be elucidated^25,26^. Here we used AP-TNFα to detect the expression of its receptors on what appeared to be immune infiltrate cells in an arthritis animal model; we were subsequently able to identify these specific immune infiltrates as macrophages. Using sequential staining with AP-TNFα and soluble receptor of IL-20R2, we recently discovered that TNFα signaling converges with that of the IL-20 subfamily of cytokines in the pathogenesis of RA^23^. For other more poorly understood TNF ligand-receptor signaling pathways such as the immune- costimulators which are believed to be promising immuno-oncology therapeutic targets^27,28^ – including 4-1BBL/4-1BB, OX-40L/OX-40, GITRL/GITR, CD30L/CD30, LIGHT/HVEM and CD70/CD27 – AP-tagged TNFSF ligands could similarly be used to help elucidate their differential expression, signaling and functional roles in immune-surveillance of cancer.

In summary, the use of AP-tagged TNFSF cytokines is a simple, sensitive and accurate approach for functional detection of the expression of corresponding TNF family of receptors *in vitro* and *in vivo*. After many years of relying on antibodies as the predominant approach for receptor detection, AP-Tag offers an additional tool that may be used in lieu of antibodies for functional detection of TNFRSF receptors and identification of the cell types involved in receptor signaling during disease onset and progression.

## Methods

### Ethics Statement

All human tissues were collected from patients at the West China Second University Hospital under approved guidelines by the Institutional Review Board of West China Hospital, Sichuan University and informed consent was obtained from all patients, or their relatives. All animal experiments were approved by the Institutional Animal Care and Use Committee of West China Hospital, Sichuan University, and the experimental procedures were performed in accordance with the approved guidelines.

### Construction and expression of AP-Tagged TNF family of cytokines and receptor-Fc fusions

cDNAs encoding the mature human TNFα, TRAIL and RANKL were gene synthesized after codon optimization for CHO cell expression (GeneScript, Nanjing, China). The cDNAs were cloned into pAP-TAG4-D expression vector (GenHunter Corporation, Nashville, TN) at *HindIII-BglII* sites for in-frame fusion at the C-terminus of AP. cDNA encoding soluble human TNFRII, DR5 and RANK were gene synthesized after codon optimization for CHO cell expression (GeneScript, Nanjing, China) and cloned into pGH-Fc-D expression vector (GenHunter Corporation, Nashville, TN) at *HindIII-BglII* sites for in-frame fusion at the N-terminus of human IgG1 Fc. The resulting plasmids were transfected into GH-CHO (*dhfr-*) cells, selected without hypoxanthine thymine (HT) (Invitrogen), and stepwise gene amplified with increasing concentration of MTX (Sigma) for high titer expression of each fusion proteins under serum free culture using SFM4CHO medium (HyClone, Logan, UT). The conditioned serum free media containing AP alone and AP fusion proteins were collected and used directly for subsequent experimentation. Receptor Fc fusion proteins were purified from the conditioned media via mAbSelect protein A (GE Biosciences) chromatography.

### Cell lines and culture

GH-CHO (*dhfr*-) Chinese hamster ovary (CHO) cell line was obtained from GenHunter Corporation (Nashville, TN). WEHI 164 mouse fibrosarcoma cell line, HCT116 colon cancer cell line and pancreatic cancer cell lines AsPC-1, BxPC-3 and Capan-2 were from the American Type Culture Collection (ATCC). CHO cells were maintained in IMDM (HyClone, Logan, UT) and adapted to serum free culture in SFM4CHO (HyClone, Logan, UT) for production of AP fusion and Fc fusion proteins. WEHI 164 and pancreatic cancer cell lines were maintained in RPMI 1640 (HyClone, Logan, UT) supplemented with 10% fetal bovine serum (HyClone, Logan, UT) and 1% penicillin-streptomycin (HyClone, Logan, UT) at 37 °C with 5% CO_2_.

### Western blot and ligand affinity blot analysis

Conditioned media of AP or AP-tagged TNF ligands (both at 1 U/mL, about 10 nM according to specific activity of AP^15^) were detected by Western blot on a 10% SDS-PAGE using anti-AP rabbit polyclonal antibody (GenHunter) followed by goat anti-rabbit IgG-HRP (Southern Biotech) to verify each AP-tagged ligands. Affinity blot analysis of TNF receptors was carried out using AP assay reagent S (GenHunter) following the manufacturer’s instructions as previously described^29^. Two μg of each purified recombinant Fc tagged receptor was separated on a 10% SDS-PAGE under non-reducing condition and either visualized by Coomassie Blue staining or transferred to PVDF membranes. After blocking with 5% fat-free milk in PBS, the membranes were incubated for 1 hour with conditioned media of either AP or AP-TNFα, AP-TRAIL and AP-RANKL followed by visualization with AP Assay Reagent S (GenHunter Corporation). The AP activity of all conditioned media were kept equal at 1 U/mL.

### ELISA

Solid phase Sandwich ELISA assays were performed to quantitatively detect recombinant TNF receptor-ligand binding activity under native conditions. Recombinant receptor-Fc proteins at 0.1 μg/mL were added into each well of 96-well ELISA plates (Maxsorp, Corning) which were previously coated with protein A. Following 1 hr of ligand binding with AP or AP-TNFα/-TRAIL/-RANKL conditioned media (all at 1 U/mL), the receptor binding activities were detected with AP substrate, AP assay reagent A (GenHunter), following the manufacturer’s instructions as previously described^29^. ELISA plates were washed with PBS three times between each step and once at the final step before adding AP substrate with AP wash buffer (10 mM Tris, 50 mM KCl, 1.5 mM MgCl_2_, 0.01‰ gelatin, pH 8.4).

### Bioassays

The biological activities of AP labeled TNFα and TRAIL were assessed by a MTT tetrazolium (Sigma) cytotoxicity assay with rhTNFα^30^ or rhTRAIL (R&D Systems) as positive controls, respectively. In brief, WEHI-164 cells with 500 ng/mL ActD (Sigma) and TRAIL-sensitive BxPC-3 were seeded in 96-well plates at a cell density of 5 × 10^5^ and 2.5 × 10^5^/well in 50 μL medium, respectively. The same volume of 2-fold serial dilutions of either unlabeled or AP-tagged TNFα and TRAIL in RPMI 1640 were added to the respective target cells. After 20 hr of incubation at 37 °C, 0.5 mg/mL MTT was added and further incubated for 4 hr. After aspirating the media from the wells, 100 μL of DMSO was added to dissolve Formazan crystal. The absorbance at 490 nm was recorded. The specific activity of each cytokine was determined as U/micromole for comparison of each pair of AP-tagged and untagged ligand.

### Cell surface receptor binding assay

Quantitative cell surface receptor binding studies were carried out as described previously^31^ with some modifications. Briefly, cells were seeded at 5 × 10^5^/well in 6-well plates in duplicates. After cells reached confluence, medium was removed until 100 μL remained in each well, and then plates were steamed in a boiling water bath for 10 min with open lids. After removing remaining medium, 1 mL of either the AP fusion protein with or without 100 μg/mL unlabeled rhTNFα and rhTRAIL, or AP alone containing media (all at 1 U/mL) was added into each well of cells for 1.5 hr. After removing the AP containing media, the plates were washed 5 times with HBHA wash buffer (1 × Hanks buffer, 0.5 g/L BSA, 1 M HEPES), cells were lysed in cell lysis buffer (1% Triton X100, 10 mM Tris, pH 8.0) and supernatant were collected for AP activity assay using reagent A (GenHunter Corporation, Nashville, TN) following the manufacturer’s instructions. For saturation binding assays, WEHI-164 and BxPC-3 cells were allowed to bind increasing amount of AP-TNFα or AP-TRAIL ranging from 0 to 40 nM based on the specific activity of AP as described previously^15^. Receptor specific ligand binding was determined by subtracting the background signals from blank medium incubated cells and analyzed by GraphPad Prism. Each data point was determined in duplicate.

### Histological analysis and tissue *in situ* ligand staining

Tissues from human colon, breast, lung and pancreatic cancers, the paws from mouse CIA model and skin samples from the back of mouse IMQ model and psoriasis patient were fixed in 10% neutral buffered formalin, paraffin-embedded, and sectioned at 5μm and stained with H&E. *In situ* ligand staining studies were performed essentially as previously described^32^. The sections were incubated at 65 °C for 90 min to de-wax before being rehydrated and incubated with either 1 U/mL of AP or AP-TRAIL and AP-TNFα, mixture of AP-TRAIL and rhTRAIL at 10 μg/mL or AP-TNFα and rhTNFα at 10 μg/mL for 90 min. After washing 3 times with HBHA wash buffer and fixing with fix reagent (60% acetone, 3% formaldehyde and 20 mM HEPES, pH 7.5), the sections were stained with AP assay reagent S.

## References

[1] Locksley RM, Killeen N, Lenardo MJ (2001). The TNF and TNF receptor superfamilies: integrating mammalian biology. Cell.

[2] Bodmer J-L, Schneider P, Tschopp J (2002). The molecular architecture of the TNF superfamily. Trends Biochem. Sci..

[3] Naismith JH, Sprang SR (1998). Modularity in the TNF-receptor family. Trends Biochem. Sci..

[4] Aggarwal BB (2003). Signalling pathways of the TNF superfamily: a double-edged sword. Nat. Rev. Immunol..

[5] Croft M, Benedict CA, Ware CF (2013). Clinical targeting of the TNF and TNFR superfamilies. Nat. Rev. Drug Discov..

[6] van Schouwenburg PA (2013). Adalimumab elicits a restricted anti-idiotypic antibody response in autoimmune patients resulting in functional neutralisation. Ann. Rheum. Dis..

[7] Nanda, K. S., Cheifetz, A. S. & Moss, A. C. Impact of antibodies to infliximab on clinical outcomes and serum infliximab levels in patients with inflammatory bowel disease (IBD): a meta-analysis. *Am. J. Gastroenterol*. **108**, 40–47, quiz 48 (2013). 10.1038/ajg.2012.363 PMC3561464 23147525

[8] Yagita H, Takeda K, Hayakawa Y, Smyth MJ, Okumura K (2004). TRAIL and its receptors as targets for cancer therapy. Cancer Sci..

[9] Ashkenazi A, Herbst RS (2008). To kill a tumor cell: the potential of proapoptotic receptor agonists. J. Clin. Invest..

[10] Dimberg LY (2013). On the TRAIL to successful cancer therapy? Predicting and counteracting resistance against TRAIL-based therapeutics. Oncogene.

[11] Jin Z, McDonald ER, Dicker DT, El-Deiry WS (2004). Deficient tumor necrosis factor-related apoptosis-inducing ligand (TRAIL) death receptor transport to the cell surface in human colon cancer cells selected for resistance to TRAIL-induced apoptosis. J. Biol. Chem..

[12] Burry RW (2011). Controls for immunocytochemistry: an update. J. Histochem. Cytochem. Off. J. Histochem. Soc..

[13] Tartaglia LA (1995). Identification and expression cloning of a leptin receptor, OB-R. Cell.

[14] Zhang R, Tan Z, Liang P (2000). Identification of a novel ligand-receptor pair constitutively activated by ras oncogenes. J. Biol. Chem..

[15] Flanagan JG, Leder P (1990). The kit ligand: a cell surface molecule altered in steel mutant fibroblasts. Cell.

[16] Cheng HJ, Flanagan JG (1994). Identification and cloning of ELF-1, a developmentally expressed ligand for the Mek4 and Sek receptor tyrosine kinases. Cell.

[17] Horiuchi K (2007). Cutting edge: TNF-alpha-converting enzyme (TACE/ADAM17) inactivation in mouse myeloid cells prevents lethality from endotoxin shock. J. Immunol. Baltim. Md 1950.

[18] Zheng Y, Schlondorff J, Blobel CP (2002). Evidence for regulation of the tumor necrosis factor alpha-convertase (TACE) by protein-tyrosine phosphatase PTPH1. J. Biol. Chem..

[19] Espevik T, Nissen-Meyer J (1986). A highly sensitive cell line, WEHI 164 clone 13, for measuring cytotoxic factor/tumor necrosis factor from human monocytes. J. Immunol. Methods.

[20] Zhou Y, Bowie JU (2000). Building a thermostable membrane protein. J. Biol. Chem..

[21] Giver L, Gershenson A, Freskgard PO, Arnold FH (1998). Directed evolution of a thermostable esterase. Proc. Natl. Acad. Sci. USA.

[22] Rabbani G, Kaur J, Ahmad E, Khan RH, Jain SK (2014). Structural characteristics of thermostable immunogenic outer membrane protein from Salmonella enterica serovar Typhi. Appl. Microbiol. Biotechnol..

[23] Liu, X. *et al*. A Broad Blockade of Signaling from the IL-20 Family of Cytokines Potently Attenuates Collagen-Induced Arthritis. *J. Immunol. Baltim. Md 1950*, doi:10.4049/jimmunol.1600399 (2016). 10.4049/jimmunol.1600399 27619991

[24] Kristensen M (1993). Localization of tumour necrosis factor-alpha (TNF-alpha) and its receptors in normal and psoriatic skin: epidermal cells express the 55-kD but not the 75-kD TNF receptor. Clin. Exp. Immunol..

[25] Brenner D, Blaser H, Mak TW (2015). Regulation of tumour necrosis factor signalling: live or let die. Nat. Rev. Immunol..

[26] Kalliolias GD, Ivashkiv LB (2016). TNF biology, pathogenic mechanisms and emerging therapeutic strategies. Nat. Rev. Rheumatol..

[27] Bartkowiak T, Curran MA (2015). 4-1BB Agonists: Multi-Potent Potentiators of Tumor Immunity. Front. Oncol..

[28] Linch SN, McNamara MJ, Redmond WL (2015). OX40 Agonists and Combination Immunotherapy: Putting the Pedal to the Metal. Front. Oncol..

[29] Wang M, Tan Z, Zhang R, Kotenko SV, Liang P (2002). Interleukin 24 (MDA-7/MOB-5) signals through two heterodimeric receptors, IL-22R1/IL-20R2 and IL-20R1/IL-20R2. J. Biol. Chem..

[30] Luo D (2016). High Level Expression and Purification of Recombinant Proteins from Escherichia coli with AK-TAG. PloS One.

[31] Flanagan JG (2000). Alkaline phosphatase fusions of ligands or receptors as *in situ* probes for staining of cells, tissues, and embryos. Methods Enzymol..

[32] He M, Liang P (2010). IL-24 transgenic mice: *in vivo* evidence of overlapping functions for IL-20, IL-22, and IL-24 in the epidermis. J. Immunol. Baltim. Md 1950.

