# Author Correction: Functional Detection of TNF Receptor Family Members by Affinity-Labeled Ligands

**DOI:** 10.1038/s41598-018-24986-9

**Published:** 2018-04-30

**Authors:** Yang Xu, Lingmo Chang, Anliang Huang, Xiaojun Liu, Xinyu Liu, Hong Zhou, Joshua G. Liang, Peng Liang

**Affiliations:** 1 Department of Biochemistry & Molecular Biology, College of Life Sciences, Sichuan University, Chengdu, 610064 China; 2 Laboratory for Gene and Cell Therapy, Sichuan University, Chengdu, 610064 China; 3 Departmentof Biochemistry and Molecular Biology, Southwest Medical University, Luzhou, 646000 China; 4 Clover Biopharmaceuticals, Chengdu, 610041 China

Correction to: *Scientific Reports* 10.1038/s41598-017-06343-4, published online 31 July 2017

In the original version of this Article, Affiliation 1 was incorrectly listed as ‘Department of Biochemistry & Molecular Biology, School of Life Sciences, Sichuan University, Chengdu, 610064, China’. The correct affiliation is listed below:

Department of Biochemistry & Molecular Biology, College of Life Sciences, Sichuan University, Chengdu, 610064, China

This error has now been corrected in the PDF and HTML versions of the Article.

